# Transcription Factors in Cartilage Homeostasis and Osteoarthritis

**DOI:** 10.3390/biology9090290

**Published:** 2020-09-14

**Authors:** Margot Neefjes, Arjan P. M. van Caam, Peter M. van der Kraan

**Affiliations:** Experimental Rheumatology, Radboud University Medical Centre, Geert Grooteplein 28, 6525 GA Nijmegen, The Netherlands; margot.neefjes@radboudumc.nl (M.N.); arjan.vancaam@radboudumc.nl (A.P.M.v.C.)

**Keywords:** osteoarthritis, cartilage, transcription factors, gene expression analysis, transcriptome, transcriptional regulation

## Abstract

Osteoarthritis (OA) is the most common degenerative joint disease, and it is characterized by articular cartilage loss. In part, OA is caused by aberrant anabolic and catabolic activities of the chondrocyte, the only cell type present in cartilage. These chondrocyte activities depend on the intra- and extracellular signals that the cell receives and integrates into gene expression. The key proteins for this integration are transcription factors. A large number of transcription factors exist, and a better understanding of the transcription factors activated by the various signaling pathways active during OA can help us to better understand the complex etiology of OA. In addition, establishing such a profile can help to stratify patients in different subtypes, which can be a very useful approach towards personalized therapy. In this review, we discuss crucial transcription factors for extracellular matrix metabolism, chondrocyte hypertrophy, chondrocyte senescence, and autophagy in chondrocytes. In addition, we discuss how insight into these factors can be used for treatment purposes.

## 1. Introduction

Osteoarthritis (OA) is the most common degenerative joint disease, and it is characterized by progressive articular cartilage loss, osteophyte formation, and subchondral bone remodeling. In addition, OA is often accompanied by synovial inflammation. Ultimately, OA can cause disability, which greatly and negatively impacts the quality of life of patients [1]. Treatment options for OA are very limited as there are, to date, no disease-modifying drugs available [2].

Articular cartilage is the connective tissue covering the end of bones in articular joints. This tissue is of crucial importance in facilitating smooth and frictionless movement and acts as a shock absorber upon movement. It is a special type of tissue as it is non innervated and does not contain blood vessels for the supply of oxygen and nutrients [3,4]. In addition, there is only one highly specialized cell type present, i.e., the chondrocyte [3,5]. Chondrocytes are essential for constructing, maintaining, and repairing the extracellular matrix (ECM) [6], which makes up for 98% of articular cartilage [5], and they do so by balancing the production of major matrix components, like collagen type II (COL2A1) and aggrecan (ACAN), with that of ECM-degrading enzymes, such as matrix metalloproteinases (MMPs) and a disintegrin and metalloproteinase with thrombospondin motif (ADAMTSs). Aberrant chondrocyte function contributes to OA, and numerous homeostatic processes are disturbed in OA, including matrix maintenance and autophagy. Aberrant chondrocyte function can be caused by an altered phenotype; in OA, amongst others, both a hypertrophy-like phenotype, in which the cells resemble hypertrophic chondrocytes in the growth plate, and a senescence-associated secretory phenotype (SASP), in which chondrocytes produce various proinflammatory cytokines, can be observed [7,8].

Ultimately, this change in chondrocyte function and phenotype observed during OA is due to the intra- and extracellular signals that the chondrocyte receives and integrates into gene expression over time. The integration of these different signals runs via modulation of transcription factor (TF) activity, the key proteins that regulate gene expression. A disrupted amount or activity of TFs is the cause of various diseases, including cancer [9,10], developmental disorders [11], cardiovascular disease [12], and autoimmune diseases such as systemic lupus erythematosus [13,14]. In this review, we will explore the role of TFs in OA (development) by addressing how the most important ones regulate ECM metabolism, chondrocyte hypertrophy, chondrocyte senescence, and autophagy on a transcriptional level. Furthermore, the potential of the transcription factor profile to help stratify patients into different subtypes and the value of this for personalized medicine and treatment options will be discussed.

## 2. Transcription Factors

TFs are DNA-binding proteins that regulate gene transcription [15,16]. This makes them key cellular components that determine how a cell reacts to both intra- and extracellular signals by converting these signals into gene expression, and, ultimately, TFs control crucial biological processes such as cell cycle progression, maintenance of metabolic balance, and cellular differentiation [17,18]. Many TFs are present in the human genome; estimations range from 500 to 2600 TFs [15,19,20]. Various types of TFs exist. To begin, there are those that are obligatory for any gene expression, such as transcription factor II A and transcription factor II B, because they are part of the transcription initiation complex [21]. Then, there are those TFs that determine cellular lineage, the so-called master regulators (MRs) [22], which control the expression of hundreds of lineage-specific genes. These MRs typically work by regulating histone-modifying enzymes to control the epigenetic state of chromatin and regulate enhancer and promoter activity to ensure a gene expression profile fitting for the cell type [23]. An example of such an MR is myoblast determination protein (MYOD), which by itself can drive mesenchymal cells to differentiate into myoblasts [24]. The genes that are made accessible by these MRs are subject to regulation by the third class of TFs, the classical transcription factors that bind to the promoter or any other regulatory element and (directly) regulate target gene transcription.

**Figure 1:**
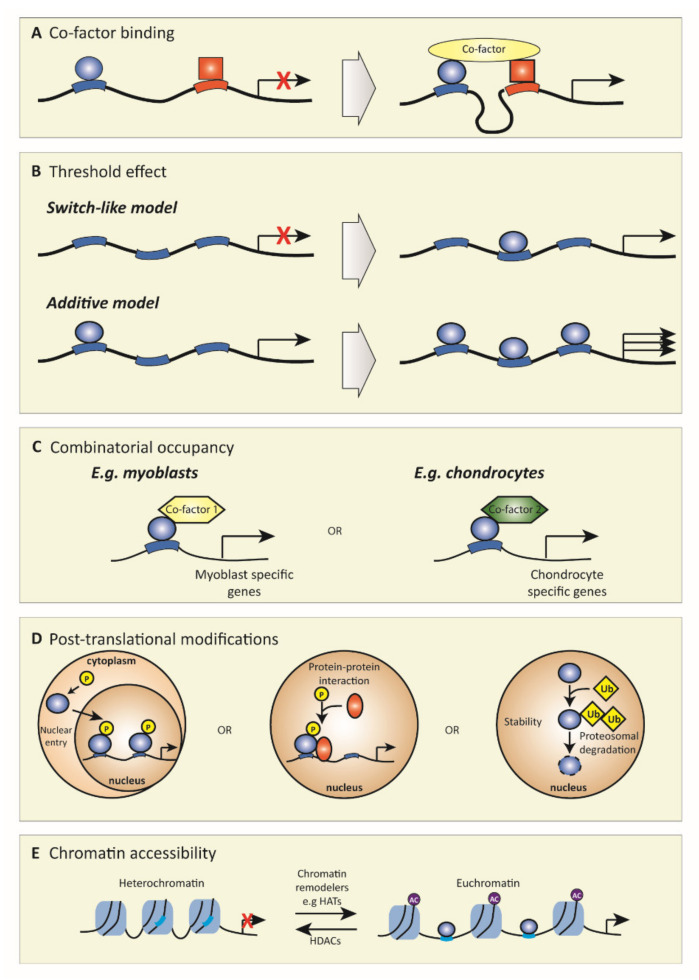
A schematic depiction of different mechanisms of transcription factor function. Models of how transcription factors can affect transcription by (**A**) cofactor binding, (**B**) threshold effect, (**C**) combinatorial occupancy, (**D**) post-translation modifications, and (**E**) chromatin accessibility. (**A**) Some transcription factors need cofactors to activate transcription. For example, cofactor binding can create a looping of the DNA, which brings transcription factors that are normally far apart into close proximity, which results in activated transcription. (**B**) For some genes, transcription is an on/off switch where, in the absence of transcription factors, no transcription occurs, while above a certain concentration binding of transcription factors results in transcription (switch-like model). For other genes, binding of more transcription factors results in higher gene expression (additive model). (**C**) The same transcription factor can induce different responses based on its interaction partners (combinatorial occupancy). For example, in myoblasts, transcription factor 1 interacts with cofactor 1 to express myoblast specific genes, while in chondrocytes, this same transcription factor interacts with cofactor 2 to express chondrocyte specific genes. (**D**) Post-translational modification is very important for the function of transcription factors, e.g., they can cause nuclear entry of transcription factors that are otherwise rendered in the cytoplasm or they can affect protein–protein interactions, and they have an important role in protein stability and turnover. (**E**) Chromatin accessibility determines if transcription factor binding sites are available for transcription factors to bind. Heterochromatin, a very dense nucleosome structure, is not accessible for transcription factors to bind, whereas euchromatin, a less dense nucleosome structure, is available for transcription factor binding. HATs = histone acetyl transferases; HDACs = histone deacetylases.

To function, TFs need to bind DNA. For this, TFs recognize highly conserved unique sequences of nucleotides of approximately 6 to 12 base pairs long [25]. Such TF binding sites can be located close to the transcription start site (TSS), but there are also so-called *cis*-regulatory elements that can be located hundreds and even thousands of base pairs from the TSS, such as insulators, enhancers, and silencers [25]. There are various mechanisms how TF function can be modulated to correctly coordinate specific gene expression, which is fundamental to cellular function (Figure 1). First, TF function can greatly depend on the interaction with other TFs or cofactors (factors that regulate transcription but do not have DNA-binding capacity) [26]. For example, whether distally located regulatory elements interact (or not) with the proximal promoter can depend on looping mechanisms that can only occur through certain cofactor binding (Figure 1A) [27]. Furthermore, the binding affinity of TFs to DNA can be subject to modulation by recruitment of other factors, e.g., low-affinity can change into high-affinity via cofactor binding [28]. For example, recombinant RelA dimers bind to nuclear factor kappa B (NFκB) DNA sites; however, when another TF such as p53 is added, RelA DNA binding affinity increases up to 4-fold [29]. If a TF binds DNA, this does not necessarily result in gene expression; this can be threshold-dependent, i.e., binding of low levels of TF does not induce gene expression, but binding of TFs above a certain threshold concentration activates gene expression, creating a switch-like effect (Figure 1B). For example, in T-lymphocytes, the TF nuclear factor of activated T-cells (NFAT) only induces the expression of its target genes above a threshold concentration [30]. Alternatively, an additive model is also possible where activation is proportional to the concentration of the TF, as was shown for NFκB, where an increased concentration led to a proportionally increased gene transcription [31]. Another key aspect that strongly regulates TF activity is combinatorial occupancy. This mechanism depends on the interaction of TFs with other TFs or cofactors to alter their function. Thereby, the same TF can induce different responses based on its interaction partners (Figure 1C). For example, SMAD family member 3 (SMAD3), a downstream TF of transforming growth factor β (TGFβ) signaling, has many cell-type-specific effects due to interacting with different cofactors in different cell types, such as octamer-binding transcription factor 4 in embryonic stem cells and MYOD1 in myotubes [32]. Another regulatory mechanism of TF activity is post-translational protein modification (Figure 1D). Post-translational modifications can affect nuclear entry, protein–protein interactions, and protein turnover. For example, (de)phosphorylation is an important modification, i.e., the phosphorylation of the NFκB family member RelA at position Ser536 results in the interaction with the cofactor p300, enhancing the transcriptional activity, whereas lack of this phosphorylation prevents this interaction [33]. Last, chromatin accessibility also greatly affects TF function (Figure 1E). Heterochromatin, a very compact nucleosome structure, is almost not accessible for TFs, whereas euchromatin, which has a less dense structure, facilities TF binding. The conformation of chromatin is mainly determined by the histone modifications present. These different mechanisms that determine TF function are extensively reviewed in [26].

## 3. Transcription Factors in OA

The importance of a well-regulated TF network is demonstrated by the fact that errors in these mechanisms or mutations in TF binding sites result in various diseases, such as cancer and autoimmunity [10,13]. Now we will discuss the role of a selection of TFs in cartilage and OA (development). A key TF for chondrocyte phenotype and activity is SRY-BOX transcription factor 9 (SOX9) asthis TF is the MR of chondrogenesis and regulates different phases of chondrocyte development, e.g., by stimulating early mesenchymal condensation and proliferation and inhibiting chondrocyte hypertrophy and chondrocyte senescence [34,35,36]. The importance of SOX9 for cartilage development has been established both in vitro and in vivo. For example, the embryonic stem cells of *Sox9*-knockout mice cannot form cartilage [34], and inactivation of *Sox9* via the Cre/LoxP system in mouse embryos before mesenchymal condensation results in a complete absence of cartilage formation [37]. Furthermore, in humans, haploinsufficiency of *SOX9* results in a lethal skeletal malformation syndrome called campomelic dysplasia [38]. In human OA chondrocytes, *SOX9* expression is reduced compared to healthy chondrocytes [35,39,40]. Possibly, this loss of SOX9 as MR relates to the altered chromatin landscape observed in OA. A recent study using ATAC-sequencing (a technique to assess genome-wide chromatin accessibility) identified that the genome of OA chromosomes was more accessible for transcription at 1565 places and less accessible at 2885 places compared to healthy cartilage [41]. This included enhancer regions regulating important genes such as *SOX9*, *SOX11*, and many other genes involved in mesenchymal fate commitment, regulators of ossification, and chondrocyte differentiation. Furthermore, enrichment analysis of TF-binding motifs in the differentially accessible regions identified binding sites for TFs such as activator protein 1 (AP-1), CCAAT-enhancer-binding protein (C/EBP), erythroblast transformation specific (ETS), and signal transducer and activator of transcription 3 (STAT3), suggesting that these TFs might be important in regulating altered gene expression in OA. In addition, a transcriptome study reported differential expression of 93 TFs (out of a total of 865) in human knee OA cartilage compared to normal cartilage [42]. These 93 TFs included *JUN*, *EGR1*, *JUND*, *FOSL2*, *MYC*, *KLF4*, *RelA*, and FOS. Another study also identified *JUN*, *JUND*, *MYC*, and *FOS* as the four TFs that are central in regulating signaling pathways in OA knee cartilage [43].

Altered post-translational modifications may also play a role in OA [44]. TFs such as AP-1, SMAD3/4, hypoxia-inducible factor (HIF), and C/EBP are targets of post-translational modifications [45]. The importance of post-translational modifications, for example, phosphorylation in OA, is demonstrated by inhibition of p38 mitogen-activated protein kinase (MAPK), a kinase that can phosphorylate TFs in pellets from articular cartilage chondrocytes, which resulted in decreased glycosaminoglycan deposition and increased expression of hypertrophy markers [46]. Furthermore, inhibition of the p38 MAPK pathway resulted in suppressed apoptosis and decreased expression of proinflammatory cytokines in human OA chondrocytes [47]. Interestingly, increased activation of p38 MAPK was detected in human OA cartilage versus control cartilage [48]. Inflammation is also shown to influence these kinases and phosphatases in chondrocytes, suggesting that under inflammatory conditions, post-translational modification of transcription factors might be altered [49]. Altogether, these studies emphasize the crucial role of TFs in OA development, and we will now discuss the role of TFs in four crucial processes of OA: ECM metabolism, hypertrophy, SASP, and autophagy.

### 3.1. Transcription Factors Related to ECM Metabolism

**Figure 2:**
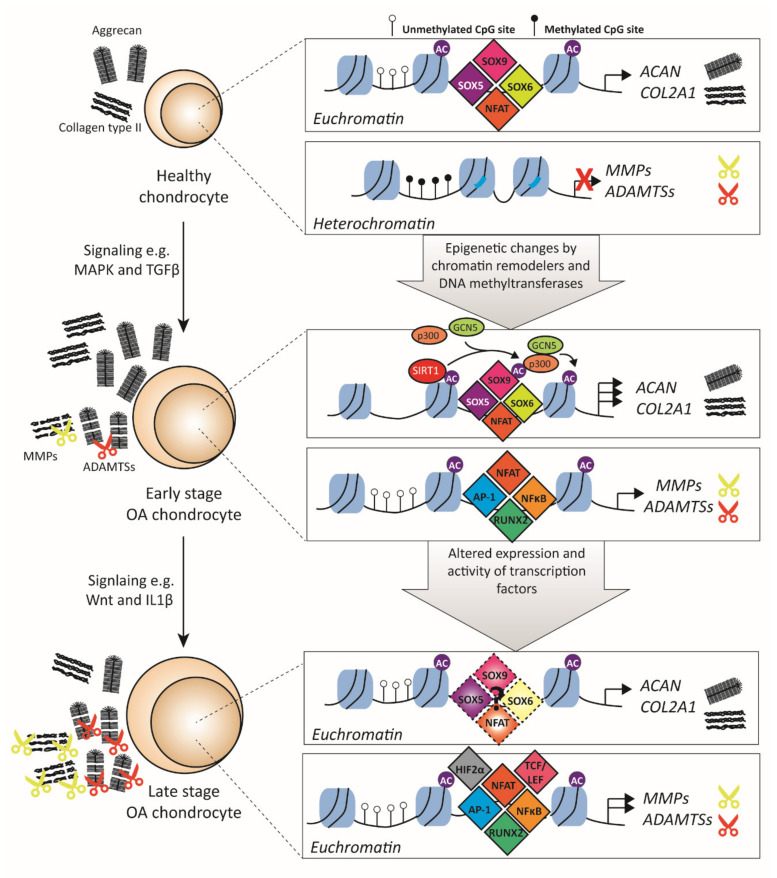
Transcription factors involved in extracellular matrix (ECM) metabolism. Model of how transcription factors are involved in the regulation of ECM production and degradation. Healthy chondrocytes synthesize ECM proteins such as ACAN and COL2A1. Important transcription factors for these genes are SOX5, SOX6, SOX9, and NFAT. In addition, in a healthy chondrocyte, matrix-degrading enzymes, such as MMPs and ADAMTSs, are expressed at a low level or not at all. These genes are silenced by epigenetic modifications. During osteoarthritis (OA), epigenetic changes occur by chromatin remodelers, resulting in either a conformation to euchromatin or heterochromatin, depending on the gene. In early OA, chondrocytes increase the production of important ECM proteins. One of the proposed mechanisms for this increase is the enhanced activation of transcription factors such as SOX9. SIRT1 can acetylate SOX9, which results in the recruitment of HAT cofactors such as p300 and GCN5, which, in turn, hyperacetylate surrounding histones. At this stage, transcription factors are recruited to the promoter regions of the ECM-degrading enzyme genes, such as AP-1, RUNX2, NFAT, and NFκB, and transcription takes place. In late OA chondrocytes, transcription levels of ECM-degrading enzymes increase, together with a loss of ECM production, as there are altered expression and function of transcription factors that are important for both these processes. Furthermore, epigenetic changes also occur, and, together, this results in the silencing of the ECM genes. MMP = matrix metalloproteinase; ADAMTS = a disintegrin and metalloproteinase with thrombospondin motif; AP-1 = activator protein 1; RUNX2 = runt-related transcription factor 2; NFAT = nuclear factor of activated T-cells; HAT = histone acetyltransferase; NFκB = nuclear factor kappa B; ACAN = aggrecan; COL2A1 = collagen type II; SOX5 = SRY-BOX transcription factor 5; SOX6 = SRY-BOX transcription factor 6; SOX9 = SRY-BOX transcription factor 9; HIF2α = hypoxia-inducible factor 2-alpha; TCF/LEF = T-cell factor/lymphoid enhancer factor.

In healthy cartilage, chondrocytes maintain the ECM by synthesizing and remodeling its components. Chondrocytes do this by balancing the production of ECM molecules with that of ECM-degrading molecules. Two major ECM protein components of articular cartilage are COL2A1 and ACAN [3], while key proteins in cartilage ECM degradation are members of the MMP and ADAMTS families. Both families consist of multiple members (19 ADAMTSs [50] and 23 MMPs members in humans [51]), but MMP3, MMP13, ADAMTS4, and ADAMTS5 are the most important ones in cartilage [52]. In OA, the balance in matrix production and degradation is disturbed, leading to loss of ECM, which is one of the main hallmarks of OA. This disturbed balance can be the result of decreased ECM deposition, increased ECM degradation, or both (Figure 2). However, in early OA, chondrocytes produce more ECM proteins, as measured by increased mRNA expression and enhanced incorporation of ^35^S-radiolabeled cysteine or methionine and sulfate in proteins and proteoglycans, respectively, in early experimental OA models [53,54], making the first option unlikely in the early stages of OA development. This makes it more likely that loss of ECM is a consequence of excessive ECM degradation caused by elevated expression of ECM-degrading enzymes such as *MMPs* and *ADAMTSs*. In healthy chondrocytes, these enzymes are expressed at a low level, which is (in part) regulated by epigenetic modifications that silence their expression, for example, via DNA methylation at CpG sites, which prevent TFs from binding to DNA [55,56]. High levels of such methylation are present in healthy chondrocytes at the promoters of *MMP3*, *MMP13*, and *ADAMTS4*, but in OA chondrocytes, many of these CpG sites are unmethylated, making these genes more accessible for transcription [55]. For example, Bui et al. demonstrated that in human OA cartilage, the amount of demethylation of the *MMP13* promotor positively correlates with increased *MMP13* expression [57]. Furthermore, *ADAMTS4* promotor demethylation was also shown in human OA knee cartilage, together with increased expression of *ADAMTS4* [58], suggesting that the loss of CpG methylation could lead to enhanced expression of *ADAMTS4*. It is not exactly clear what triggers the demethylation of these promoters, but age, changes in ion concentrations, and inflammatory cytokines are implicated in this process [57,59,60]. These factors can affect DNA methylation either by a passive mechanism, where DNA methylation is inhibited by binding of TFs during cell division, or via an active mechanism by activating DNA methyltransferases to remove methyl groups [61]. Together, these studies show that the chromatin state is crucial in determining the level of expression of genes, and, in OA, this chromatin state is altered, leading to dysregulated expression of *MMPs* and *ADAMTSs*.

After opening up the chromatin, the promoter region of *MMPs* and *ADAMTSs* is accessible for TFs to regulate their expression. Key TFs for *MMP3* and *MMP13* regulation include AP-1, runt-related transcription factor 2 (RUNX2), NFκB, HIF2α, and T-cell factor/lymphoid enhancer factor (TCF/LEF) [62,63,64], whereas *ADAMTS4* and *ADAMTS5* expression strongly respond to NFAT, RUNX2, SOX4, SOX11, and NFκB [65,66]. In OA, multiples of these TFs are differently expressed. For example, expression of *AP-1*, which is a dimeric TF primarily composed of FOS and JUN proteins, has been shown to be increased in knee OA cartilage [42,43]. Inhibition of AP-1 activity in vitro by the use of the small molecule inhibitor T-5224 inhibited MMP3 and MMP13 production in SW1353 chondrocytes [67]. In vivo, the use of T-5224 in the destabilization of the medial meniscus (DMM) experimental OA mouse model inhibited the progression of cartilage degeneration by inhibiting MMP13 expression [68]. Besides *AP-1*, *SOX4* and *SOX11* mRNA levels are also increased in human OA cartilage compared to nonosteoarthritic cartilage [66,69]. Overexpression of SOX4 or SOX11 in mouse cartilage resulted in enhanced cartilage destruction, with increased ADAMTS5 and MMP13 expression [66]. However, both SOX4 and SOX11 have not been extensively studied in OA, and no deletion or inhibition studies have been performed to investigate the potential therapeutic effect hereof. NFκB, also a dimer TF, can be composed of five different subunits—NFκB1 (p105/p50), NFκB2 (p100/p52), RelA (p65), RelB, and c-Rel—and also regulates ECM-degrading enzyme expression [70]. For example, knockdown of p65 in a rat model of OA (resecting medial collateral ligament and the medial meniscus model) via intra-articular small interfering RNA (siRNA) delivery resulted in decreased degradation of cartilage compared to the vehicle-treated group [71]. Furthermore, IκB kinases (IKK) are important for the function of the NFκB family as these kinases regulate the release of NFκB from inhibitory-binding proteins in the cytoplasm, resulting in translocation to the nucleus and activation. In vitro, selective inhibition of IKK by BMS-345541 resulted in decreased expression of interleukin (IL)1β-induced expression of *MMP13* and *ADAMTS5* in human articular chondrocytes [72]. Furthermore, the administration of the IKK inhibitor in a surgically-induced OA mouse model (resecting medial collateral ligament and the medial meniscus model) resulted in significantly less cartilage destruction compared to the vehicle control group, indicating that TFs of the NFκB family regulate cartilage degradation. TFC/LEF TFs are also implicated in regulating matrix-degrading enzymes. Of the four family members (TCF1, TCF3, TCF4, and LEF1), TCF4 is most abundantly present and elevated in human OA cartilage compared with healthy cartilage [73]. Overexpression of TCF4 in human chondrocytes induced the expression of *MMP1*, *MMP3*, and *MMP13*, whereas knockdown of TCF4 resulted in decreased *MMP1* and *MMP13* mRNA levels and had no effect on *MMP3* levels. TCF/LEF TFs are mainly activated by Wnt signaling, and a small molecule Wnt inhibitor that inhibits TCF/LEF activity (SM04690) has been shown to alleviate cartilage destruction in an OA rat model by decreased expression of *Mmp1*, *Mmp3*, *Mmp13*, and *Adamts5* [74]. A clinical phase 1 study with the same small molecule inhibitor resulted in a positive effect on OA pain and function [75].

As mentioned, in the initial stage of OA, chondrocytes show increased production of ACAN and COL2A1 [76]. Both *ACAN* and *COL2A1* are regulated by the so-called SOX trio (i.e., SOX5, SOX6, and SOX9) [77]. In human knee articular cartilage of early OA patients, increased expression of *COL2A1* and *ACAN* was observed, together with increased *SOX9* expression, while the loss of *SOX9* at later stages of OA was correlated with the loss of both *COL2A1* and *ACAN* expression [78]. Besides *SOX9*, *SOX6* was also downregulated in damaged knee OA cartilage compared to undamaged cartilage [69,79]. A correlation analysis study demonstrated strong correlations between loss of TFs *SOX5* and *SOX9* and decreased expression of *COL2A1* in knee OA chondrocytes, whereas loss of *SOX6* was highly correlated with less *ACAN* expression [80]. Together, these studies suggest that the levels of the SOX trio play a crucial role in *COL2A1* and *ACAN* expression.

The function of SOX9 is not only dependent on its expression levels but also on its post-translational modifications. For example, it was shown that phosphorylation of SOX9 by cAMP-dependent protein kinase A (PKA) increased activity of a *COL2A1* promoter reporter by more efficient DNA binding of this TF in fibroblastic COS7 cells [81]. Thus, (increased) phosphorylation of SOX9 could explain the enhanced expression of *COL2A1* and *ACAN* in the early stages of OA; however, whether this happens in OA chondrocytes is not known. Another post-translational modification that affects SOX9 function is (de)acetylation. In human chondrocytes, the histone deacetylase (HDAC) sirtuin 1 (SIRT1) has been shown to bind to SOX9 and affect the acetylation status of SOX9. SIRT1 activity results in the recruitment of different cofactors such as PGC-1α, p300, and GCN5 to SOX9, and this has been linked to increased *COL2A1* expression [82]. In human OA chondrocytes, elevated SIRT1 protein expression was measured [83], and overexpression of SIRT1 results in increased *COL2A1* expression [84]. However, Fujita et al. demonstrated that SIRT1 expression levels decrease with the progression of human OA [85]. Besides (de)phosphorylation and (de)acetylation, SOX9 can also be ubiquitylated, which causes protein degradation. The ubiquitin ligase E6-AP is known to be able to ubiquitinate SOX9 in chondrocytes, and it was demonstrated that E6-AP deficient mice have elevated levels of SOX9 in their chondrocytes [86]; however, whether these mice are protected against OA development due to elevated levels of SOX9 has not been investigated yet. Furthermore, expression analysis of E6-AP and SOX9 revealed that SOX9 was present in resting, proliferating, and prehypertrophic chondrocytes, but was absent in hypertrophic chondrocytes, whereas the E6-AP was highly expressed in hypertrophic chondrocytes but absent from prehypertrophic chondrocytes [86]. Together, these studies indicate that altered post-translational modification may lead to aberrant SOX9 function in OA chondrocytes.

Besides the SOX trio, other TFs such as NFAT, paired box (PAX), SMAD3, specificity protein 1 (SP1), and glioma-associated oncogene (GLI) are also important for the transcriptional regulation of cartilage ECM proteins [77,87,88,89]. Loss (or gain) of these factors might cause impaired expression of *ACAN* and *COL2A1*. For example, articular chondrocytes of *Nfat1* KO mice showed a reduction of *Acan* and *Col2a1* expression compared to articular chondrocytes of WT mice [90]. Forced expression of NFAT1 in these cells resulted in the rescue of *Acan* and *Col2a1* expression. Additionally, family members of the SP family are involved in *COL2A1* expression, such as SP1 and SP3, but these factors have opposite roles. It was shown that in rabbit articular chondrocytes, SP1 can activate *Col2a1* expression, whereas, in the presence of SP3, this activation is lost [89]. Furthermore, it was shown that the ratio of SP3/SP1 is altered during dedifferentiation of chondrocytes, with a pronounced loss of SP1 [91]. This increased SP3/SP1 ratio can be partly attributed to the effect of proinflammatory cytokines, as in rabbit articular chondrocytes it was shown that IL1β inhibited *Col2a1* expression by increasing the SP3/SP1 ratio [92], indicating that this disturbed ratio may also play a role in decreased *COL2A1* expression in OA, as proinflammatory cytokines are present in OA. The growth factor TGFβ, which signals via the SMAD TFs, induces the expression of *COL2A1* and *ACAN* [93]. The knockdown of SMAD3 or SMAD4 during chondrogenesis in bone-marrow-derived mesenchymal stem cell pellets resulted in inhibition of both *COL2A1* and *ACAN* expression [94]. In both aging and OA cartilage, SMAD3 expression is decreased [95]. Furthermore, another TGFβ family member, bone morphogenetic protein (BMP), signals via the TF complex SMAD1/5/9. Chondrocyte-specific knockdown of both SMAD1 and SMAD5 in mice resulted in a reduction of COL2A1 deposition, while ACAN protein levels were not altered [96]. In conclusion, both ECM production, as well as ECM degradation, is altered in OA by altered TF expression and function.

### 3.2. Transcription Factors Related to Hypertrophy

**Figure 3:**
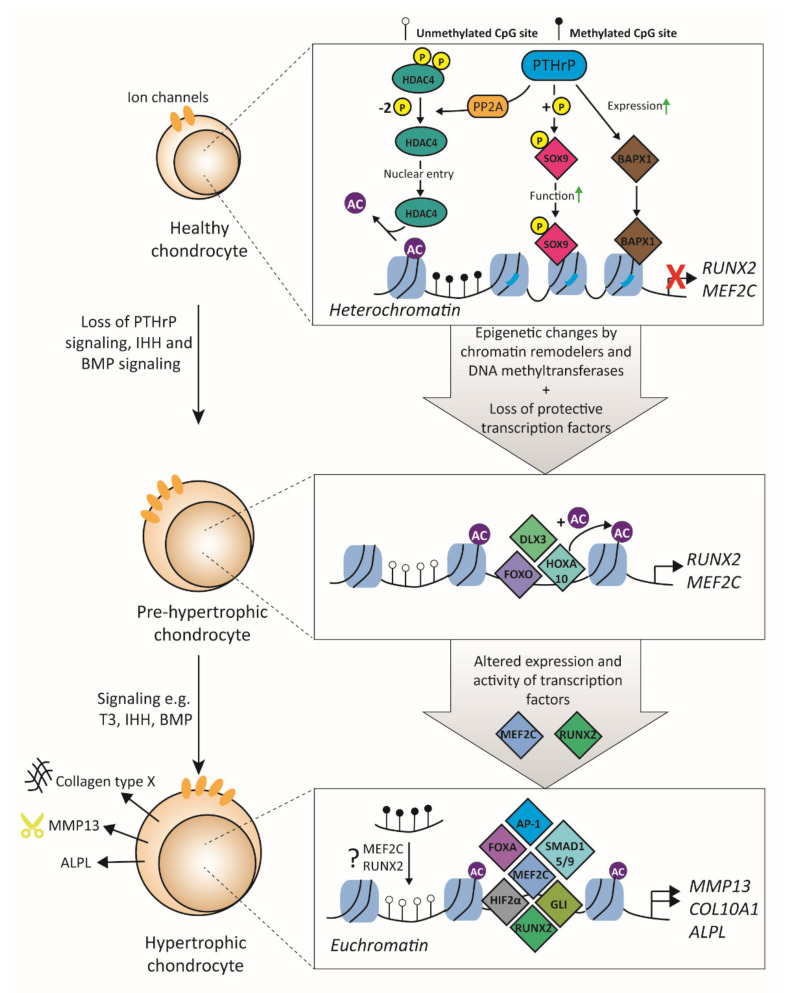
Transcription factors involved in chondrocyte hypertrophy. In a healthy chondrocyte, hypertrophy-related genes are silenced due to the compact composition of the nucleosomes and transcription factors that repress hypertrophy. PTHrP is a crucial signaling molecule that represses chondrocyte hypertrophy by increasing expression of BAPX-1, increasing SOX9 activity, and inducing nuclear translocation of HDAC4 to maintain hypoacetylation of the promoters of RUNX2 and MEF2C. In prehypertrophic chondrocytes, epigenetic changes occur by, e.g., loss of chromatin remodelers and loss of protective transcription factors against hypertrophy. This leads to increased chondrocyte volume and the opening of chromatin. TFs such as HOXA10, FOXO1, and DLX3 can bind in the promoter of RUNX2 and activate its transcription. RUNX2 and MEF2C are the MR of chondrocyte hypertrophy and regulate, in turn, with other transcription factors, the expression of hypertrophy makers such as COL10A1, MMP13, and ALPL. PTHrP = parathyroid hormone-like protein; SOX9 = SRY-BOX transcription factor 9; BAPX-1 = homeobox protein Nkx-3.2; HDAC4 = histone deacetylase 4; NFκB = nuclear factor kappa B; RUNX2 = runt-related transcription factor 2; MEF2C = myocyte-specific enhancer factor 2C; AP-1 = activator protein 1; GLI = glioma-associated oncogene; FOXA = forkhead box transcription factor class A; HIF2α = hypoxia-inducible factor 2-alpha; COL10A1 = collagen type X; MMP13 = matrix metalloproteinase 13; ALPL = alkaline phosphatase.

During OA, chondrocytes can become hypertrophic and undergo terminal differentiation [97]. Healthy chondrocytes express proteins such as Indian Hedgehog (IHH) and parathyroid hormone-like protein (PTHrP) that regulate hypertrophic differentiation via a negative feedback loop. When signaling by these factors is altered, chondrocytes become hypertrophic, obtain an increased cell size, and switch to a new genetic program, with the expression of new proteins like collagen type X (COL10A1) and MMP13 (Figure 3) [98]. In addition, hypertrophic chondrocytes actively mineralize their surroundings with protein-like alkaline phosphatase (ALPL). Multiple signaling pathways are involved in obtaining the hypertrophy-like phenotype, such as active thyroid hormone (T3), Wnt, MAP-kinases, Notch, and TGFβ signaling [98]. It is thought that before marked cartilage degradation, human chondrocytes change their morphology and volume, which are linked to the phenotypic stability of the chondrocyte [99]. The observed increased cell size at the early stages of hypertrophic differentiation is such an example. Cell size is a tightly regulated cell characteristic, and, in chondrocytes, it has been demonstrated that many factors are associated with the morphological changes that happen during hypertrophy. It is thought that reduced osmolarity in OA cartilage is one of the main causes for the increase in cell size [100]. In OA, it is shown that the transcription of ion channels is changed [101,102]. For example, acid-sensing potassium channel (*TASK-2*), epithelial sodium channel (*ENaC*), and Ca^2+^ activated chloride channel (*TMEM16*) transcription is decreased, while expression of Ca^2+^ activated potassium channels (*KCa3.1* and *KCa1.1*) increase in OA [103]. However, not much is known about which TFs regulate this aberrant expression in chondrocytes. Altered transcription of ion channels results in changes in intracellular ion concentrations, leading to an increase in cell volume. Furthermore, changes in intracellular ion concentrations can lead to the release of intracellular messengers that, in turn, can activate or repress post-translational modification enzymes [104]. These enzymes can affect the function of TFs by modulating their post-transcriptional modification sites. For example, the activation of the TF NFAT is dependent on dephosphorylation by the calcium-dependent phosphatase calcineurin [105]. In addition, other TFs are also dependent on intracellular ion concentrations, such as NFκB and CREB [106]. However, not much is known about the role of this in the terminal differentiation of chondrocytes.

Moreover, the gained expression of hypertrophy markers *COL10A1* and *MMP13* depends, firstly, on chromatin remodeling to euchromatin and, secondly, on the activation of their promoter. The remodeling of chromatin mostly depends on HDACs. HDAC4 is guided by PTHrP signaling; PTHrP causes decreased phosphorylation of HDAC4 by protein phosphatase 2 (PP2A), which results in translocation to the nucleus, where it can act as a repressor of the TFs myocyte-specific enhancer factor 2C (*MEF2C*) and *RUNX2* [107]. Both MEF2C and RUNX2 are TFs that are crucial for chondrocyte hypertrophy. Forced expression of RUNX2 induced hypertrophy in nonhypertrophic chondrocytes [108], and, similarly, expression of a superactive form of MEF2C in mice led to increased chondrocyte hypertrophy, whereas deletion of *MEF2C* in COL2A1-expressing chondrocytes in mice resulted in the absence of chondrocyte hypertrophy [109]. Of note, MEF2C is thought to be an upstream player of RUNX2. Importantly, it has been shown that HDAC4 and MEF2C have opposing roles in regulating chondrocyte hypertrophy and that the balance of these TFs is important in regulating this process. For example, the deletion of one *M*ef2c allele in *Hdac4* KO mice normalized ossification of the cartilage, while deletion of one *Hdac4* allele in *Mef2c* knockdown mice restored ossification [109]. Interestingly, HDAC4 levels decrease with age and are even further decreased in OA cartilage [110], and such loss of HDAC4 can, thus, possibly result in more RUNX2 and MEF2C activity. In contrast, increased expression of *RUNX2* is observed in OA cartilage [40], but it is not known whether *MEF2C* expression is altered in human OA cartilage. HDAC4 represses *MEF2C* and *RUNX2* transcription by a closed DNA conformation; however, when HDAC4 expression is lost during OA, DNA structure transforms from heterochromatin to euchromatin, and TFs can bind to activate transcription of *MEF2C* and *RUNX2*. HOXA10 is such a TF that binds the *RUNX2* promoter. In osteoblasts, it was shown that HOXA10 recruits histone acetyltransferases (HATs) to the *RUNX2* promoter, resulting in hyperacetylation [111]. Another TF that is important in *RUNX2* transcriptional regulation is DLX3; it was demonstrated that overexpression of DLX3 in *RUNX2* null cells resulted in induced *RUNX2* expression [112]. Other TFs that play a role in *RUNX2* expression are forkhead box transcription factor class O (FOXO) 1, DLX5, and SATB2 [113]; however, not much is known about which TFs regulate *MEF2C* expression in chondrocytes. In addition to HDACs, the methylation state of a gene is also important for either gene silencing or activation. A methylome study revealed that *MMP13* was hypomethylated in hip OA cartilage compared to control cartilage [114]. Furthermore, it has been shown that an OA-risk single nucleotide polymorphism correlated with a differently methylated region that altered expression of *RUNX2* [115]. Together, this indicates that the epigenetic state of chromatin is an important determinant of hypertrophic gene expression.

Besides the ability of PTHrP to repress prohypertrophic TFs, it can also activate (or induce) antihypertrophic TFs such as SOX9 and homeobox protein Nkx-3.2 (BAPX-1). That SOX9 is important in repressing chondrocyte hypertrophy is shown in mice with a heterozygous *Sox9* deletion, which demonstrates increased maturation of immature chondrocytes into hypertrophic chondrocytes [116]. In COS7 cells, it was shown that PTHrP can increase SOX9 phosphorylation and thereby increase its transcriptional activity [117]. Another TF that inhibits chondrocyte hypertrophy is BAPX-1, as knockdown of *BAPX-1* in human articular chondrocytes resulted in a hypertrophic phenotype shift that could be rescued by overexpression of BAPX-1 [118]. Inhibition of hypertrophy by BAPX-1 is, at least partially, related to the repression of *RUNX2* and *COL10A1* expression as BAPX-1 knockdown in mouse chondrocytes resulted in decreased expression of these two genes [119]. BAPX-1 expression is induced by PTHrP, as BAPX-1 expression was lost in *Pthrp* knockout mice, whereas stimulation of chick hypertrophic chondrocytes with PTHrP resulted in upregulation of BAPX-1 expression [120]. Another TF family that inhibits chondrocyte hypertrophy by repressing transcription of the hypertrophy markers is the SP family. The SP3/SP1 ratio is important for the regulation of gene expression, as is shown, for example, for *COL2A1* [89]. SP1 inhibits the expression of *COL10A1*, as was shown by overexpressing SP1 together with a *COL10A1* promoter reporter construct in hypertrophic cells [121]. However, in hypertrophic chondrocytes, this inhibition is lost as the SP3/SP1 ratio is higher than in nonhypertrophic chondrocytes, mainly because SP1 expression is much higher in nonhypertrophic versus hypertrophic chondrocytes [121].

After chromatin remodeling, various TFs can bind and regulate the expression of the hypertrophy markers *COL10A1* and *MMP13*. Important TFs for both genes are SMAD1/5/8, RUNX2, AP-1, NFAT, forkhead box transcription factor class A (FOXA), and HIF2α [122,123]. The importance of SMAD1/5/8 has been demonstrated by inhibition of this complex, which resulted in decreased IHH-induced mineralization by primary chondrocytes [124]. Furthermore, knockout studies have shown that both SMAD1 and SMAD5 are essential for endochondral bone formation, which relies on terminal differentiation of chondrocytes [96], indicating that they may also play a role in hypertrophy in OA. In addition, inhibition of SMAD1/5/8 by dorsomorphin after initial chondrogenic differentiation of bone-marrow-derived mesenchymal stem cells led to decreased expression of COL10A1, MMP13, and *ALPL* and completely blocked mineralization of the matrix [125]. In osteoblast differentiation, it was demonstrated that SMAD1/5/8 indirectly activates RUNX2 expression by increasing *AP-1* expression (*JUNB*), and stimulation of *JUNB*-overexpressing C2C12 cells with BMP2 resulted in higher ALPL activity than C2C12 cells without overexpressed *JUNB* [126]. Additionally, in mice hypertrophic chondrocytes, AP-1 family members such as *J*un and *Fosl2* are important as overexpression of these AP-1 family members results in higher *Col10a1* expression [127]. More importantly, JUN and FOSL2 are highly expressed in early mice hypertrophic chondrocytes [127]. Similarly, HIF2α has been shown to be crucial in inducing hypertrophy as overexpression of *Hif2α* in ATDC5 cells demonstrated increased expression of *Col10a1*, *Mmp13*, and ALPL activity [122]. Interestingly, HIF2α expression is increased in both mice and human OA cartilage, and HIF2α deficiency in mice protects against OA development in the DMM mouse model [122,128]. It has been demonstrated that HIF2α is a downstream target of the prohypertrophic T3 signaling molecule in cartilage tissue [129], explaining the induction of hypertrophy markers COL10A1, MMP13, and ALPL by T3 [130]. In addition to HIF2α, FOXA has been demonstrated to be involved in chondrocyte hypertrophy. In chicken chondrocytes, overexpression of *Foxa2* resulted in increased BMP2-induced expression of *Col10a1*, *Mmp13*, *Vegf*, and *Alpl*, whereas ablation of *Foxa2* by short hairpin RNA decreased expression of these hypertrophy markers [131]. In the DMM murine OA model, expression of FOX2A was increased in OA cartilage compared to healthy cartilage, and chondrocyte-specific deletion of *Foxa2* in the DMM model resulted in decreased cartilage breakdown [132]. In conclusion, these results provide evidence that chondrocyte hypertrophy is regulated by many different signaling pathways; however, the TFs MEF2C and RUNX2 can be considered the MRs of chondrocyte hypertrophy and, therefore, might be interesting therapeutic targets.

### 3.3. Transcription Factors Related to Cellular Senescence

**Figure 4:**
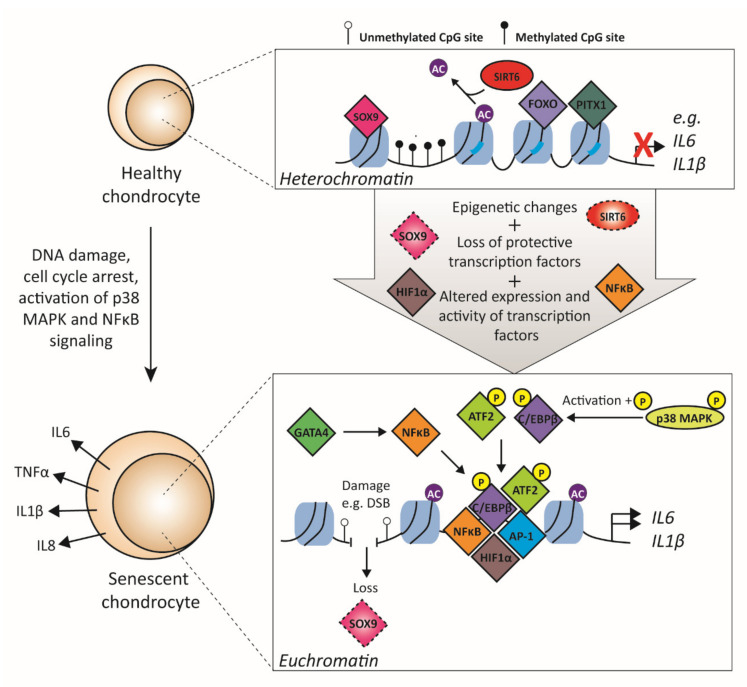
Transcription factors involved in senescence-associated secretory phenotype (SASP). In a healthy chondrocyte, senescence-related genes are silenced due to the compact composition of the nucleosomes and transcription factors that repress senescence, such as SOX9, FOXO, and PITX1. In OA development, multiple events occur, such as DNA damage, resulting in disrupted transcription factor binding. Furthermore, cells undergo cell cycle arrest, and different signaling pathways are activated. Together, this results in epigenetic changes by, e.g., loss of chromatin remodelers and loss of protective transcription factors. After the opening of chromatin, transcription factors can bind to promoter regions of SASP genes, and this results in increased transcription of these genes. In addition, the expression of these transcription factors is enhanced in OA cartilage, and their function can be enhanced by, e.g., post-translation modification by p38 MAPK. SOX9 = SRY-BOX transcription factor 9; FOXO = forkhead box transcription factor class O; PITX1 = paired-like homeodomain 1; SIRT6 = sirtuin 6; DBS = double-strand break; MAPK = mitogen-activated protein kinase; HIF1α = hypoxia-inducible factor 1-alpha; NFκB = nuclear factor kappa B; AP-1 = activator protein 1; c/EBPβ = CCAAT-enhancer-binding protein beta; ATF2 = activating transcription factor 2; IL6 = interleukin 6; IL8 = interleukin 8; IL1β = interleukin 1 beta; TNFα = tumor necrosis factor alpha.

Cellular senescence is defined by stable cell cycle arrest while the cell remains metabolically active [133]. Cellular senescence is characterized by altered morphology, chromatin structure, and gene expression pattern. Xu et al. demonstrated a role for cellular senescence in OA by injection of senescent fibroblasts in the knee joints of mice, resulting in an OA-like disease state with cartilage erosion [134]. In OA, senescent chondrocytes secrete a variety of proinflammatory cytokines, chemokines, proteases, and growth factors, and this is known as SASP. Furthermore, the expression of several proteins important in cell cycle arrest is increased in senescent cells such as p16 and p21. The mechanism of how cells go into senescence and start secreting SASP is not completely understood; however, both DNA damage and activation of p38 MAPK are thought to be crucial (Figure 4) [135]. It has been shown that DNA damage plays a role in acquiring the senescent phenotype in OA chondrocytes [136], and it is suggested that the DNA damage observed in OA chondrocytes results in a change of gene transcription activity. This might be explained by the observation that the binding capacity of TFs to damaged DNA is altered, as was experimentally shown for AP-1, NFκB, and SP1 TFs [137].

The main proinflammatory cytokines that are thought to be associated with OA development are tumor necrosis factor alpha (TNFα) and IL1β as these cytokines can stimulate the expression of a range of other proinflammatory cytokines and proteases, such as IL6, IL8, and MMPs [138]. Therefore, TNFα and IL1β are now recognized as senescence inducers. The induction of SASP is accompanied by various epigenetic changes. However, SIRT6, a deacetylase, is an important protein inhibiting cellular senescence as depletion of *SIRT6* in human chondrocytes results in the induction of cellular senescence [139]. Interestingly, SIRT6 protein levels are decreased in knee articular cartilage of OA patients compared to healthy cartilage [140]. In HeLa cells, SIRT6 interacts with the NFκB family member RelA and deacetylates H3K9 on promoters of NFκB targets genes, resulting in a more closed chromatin conformation and, therefore, decreases the accessibility of NFκB to bind to the respective promoters and activate transcription [141]. In human chondrocytes, Wu et al. demonstrated that SIRT6 protects against cellular senescence by suppressing NFκB transcriptional activity [140], suggesting that the mechanisms of deacetylating H3K9 may also occur in chondrocytes. Besides chromatin remodelers such as SIRT6, there are also TFs associated with the repression of cellular senescence and SASP. For example, SOX9 and SMAD2/3 are TFs that are known to inhibit cellular senescence by maintaining the expression of the main ECM proteins [36]. Besides these, FOXO TFs have also been shown to have a protective role by regulating inflammation and DNA repair. FOXO transcription factors are downstream targets of MAPK signaling, and phosphorylation of FOXO TFs results in its inactivation by inhibiting nuclear entry [142]. Both IL1β and TNFα increase phosphorylation of FOXO TFs and thus inhibit their activity and reduce their expression in cultured chondrocytes [143]. Furthermore, in human knee OA cartilage, higher phosphorylation of FOXO was observed compared with normal cartilage. Besides FOXO, other TFs (also the TF paired-like homeodomain 1 (PITX1)) are involved in inhibiting senescence. Inhibition of *PITX1* in nonaffected OA chondrocytes by siRNA resulted in enhanced senescence as the expression of p21, p53, and collagen type I was enhanced, while overexpression of *PITX1* resulted in decreased senescence [144]. Interestingly, PITX1 expression is lower in affected knee OA cartilage versus nonaffected knee OA cartilage [144], indicating that the protective effect of PITX1 against cellular senescence is possibly lost in OA.

An important activator of senescence is possibly GATA binding factor 4 (GATA4). In lung fibroblasts, it is shown that the TF GATA4 is crucial in cellular senescence, as ablation of *GATA4* by siRNA results in decreased cellular senescence and SASP [145]. GATA4 acts upstream of NFκB as RelA suppression decreases GATA4-induced SASP. However, not much is known about GATA4 expression in chondrocytes. MicroRNA-204 is an important upregulated factor in senescent chondrocytes, and it has been shown to be induced by GATA4 and NFκB [146], suggesting that GATA4 may also play a role in chondrocytes. Furthermore, GATA4 expression increases in multiple tissues, such as the liver and kidneys, during aging in mice, but whether this also occurs in cartilage is not known [145]. However, because OA is an age-related disease, there is a possible link. Other TFs important in regulating the expression of proinflammatory cytokines and thus supporting a SASP phenotype are AP-1, HIF1α, C/EBPβ, cAMP response element-binding protein (CREB), and SP1 [147]. The importance of HIF1α has been demonstrated; the overexpression of *HIF1α* increases *IL1B* promoter activity in human chondrocytes. [148]. In addition, HIF1α expression is reported in OA chondrocytes and OA cartilage [149]. In cancer, it has been demonstrated that the TF C/EBPβ, together with NFκB, is of major importance in the induction of SASP [150]. C/EBPβ has an autoinhibited form; however, post-translational modifications (e.g., phosphorylation) can induce a conformational change that results in the activation of C/EBPβ. In cancer, the activated RAF kinase induces such post-translational modifications and activates C/EBPβ to induce SASP production. In the chondrogenic ATDC5 cells, it was shown that overexpression of *C/ebpb*, together with *Gadd45b*, resulted in increased activation of senescent marker p21. Furthermore, C/EBPβ expression was found in senescent chondrocytes [151], suggesting that this TF might also play in role in SASP in chondrocytes. However, whether this switch to the activated form also occurs due to post-translational modifications of C/EBPβ in chondrocytes is not known. C/EBPβ is a known target of p38 MAPK, an important kinase crucial in cellular senescence that is activated during chondrocyte senescence, suggesting that induced post-translational modifications of C/EBPβ might also cause the switch to an activated TF. Additionally, other TFs important in regulating SASP inflammatory mediators, such as members of the AP-1 TF family and activating transcription factor 2 (ATF2), are subject to post-translational modifications by p38 MAPK [44]. Inhibition of p38 with the small molecule inhibitor SB203580 in rabbit articular chondrocytes resulted in a delay in senescence onset, measured by decreased senescence-associated β-galactosidase activity [152]. Furthermore, it has been shown that activated (i.e., phosphorylated) p38 levels are higher in senescent chondrocytes and that this induced the phosphorylation of ATF2, whereas this does not happen in freshly isolated chondrocytes, which have low levels of activated p38. Inactivation of p38 with a dominant-negative form resulted in both a reduction of senescence-associated β-galactosidase activity and inhibition of ATF2 phosphorylation, suggesting that the delayed onset of chondrocyte senescence is associated with post-translational modifications of ATF2. The exact mechanisms of how p38 inhibition results in decreased cellular senescence is not known, but as TFs are a main target of p38, it could occur via altered post-translational modifications of TFs.

### 3.4. Transcription Factors Related to Autophagy

Macroautophagy (often referred to as autophagy) is a mechanism in which damaged cellular constituents such as organelles and proteins are recycled via lysosomal degradation in order to maintain cellular metabolism [153]. In this process, the cell encapsulates its own components, e.g., mitochondria, in a membrane (the autophagosome), which then merges with a lysosome to recycle the components (all steps in the autophagy process and related proteins are extensively reviewed in [154]). This mechanism is induced in response to different kinds of stressors, such as nutrient starvation, hypoxia, invading organisms, and oxidative stress [155]. Activation of autophagy is a rapid process that mainly depends on post-translational protein modifications and protein–protein interactions. However, in the last decade, it became clear that especially protracted autophagic responses are mediated by a change in the transcriptional program of autophagy-related (ATG) genes [156]. Autophagy is thought to be cytoprotective and it has been suggested to be a negative regulator of inflammasome activation by degradation of inflammasome components [157,158,159]. The inflammasome regulates the immune response by maturation of proinflammatory cytokines such as IL1β and this process also contributes to OA development as inflammasome activation is enhanced in OA [160]. Autophagy activity decreases with age [161] and also plays an important role in OA development, as has been demonstrated both in vitro and in vivo. Stimulating autophagy with rapamycin (an inhibitor of mechanistic target of rapamycin kinase (mTOR) stimulating autophagy) in normal human knee articular chondrocytes resulted in decreased IL1β-induced expression of OA genes, such as *MMP13* and *ADAMTS5*, whereas inhibition of autophagy by siRNA targeting *ATG5*, an essential protein in the autophagy process, increased levels of these ECM-degrading enzymes [162]. In vivo, intra-articular injection of rapamycin in the DMM OA mouse model revealed decreased cartilage degeneration [163], whereas inhibition of autophagy with 3-methyladenine, a type III phosphatidylinositol 3-kinase (PI3K) inhibitor, in a collagenase rabbit OA model resulted in significantly aggravated cartilage degeneration [164].

Autophagy is coordinated by a large set of genes, called the ATG genes, which encode proteins that are essential in the consecutive steps of the autophagy process, such as proteins for the recruitment of cargo, formation of autophagosomes, and fusion with the lysosome. Examples of such ATG genes are Beclin-1, light chain 3 (*LC3*), and unc-51-like autophagy activating kinase 1 and 2 (*ULK1/2*) [165]. During the initial phase of OA, ATG gene expression is enhanced; for example, mRNA levels of Beclin-1 and *LC3* are increased in human articular knee OA chondrocytes compared to normal chondrocytes, whereas ATG gene expression decreases in late-stage OA [162]. This difference in kinetics is supported by an in-vivo observation: in a rabbit collagenase-induced OA model, authors demonstrated that the expression levels of autophagy markers Beclin-1 and *Lc3* were increased at 2, 4, and 6 weeks after induction of the model, whereas levels were again decreased after 8 weeks [164].

**Figure 5:**
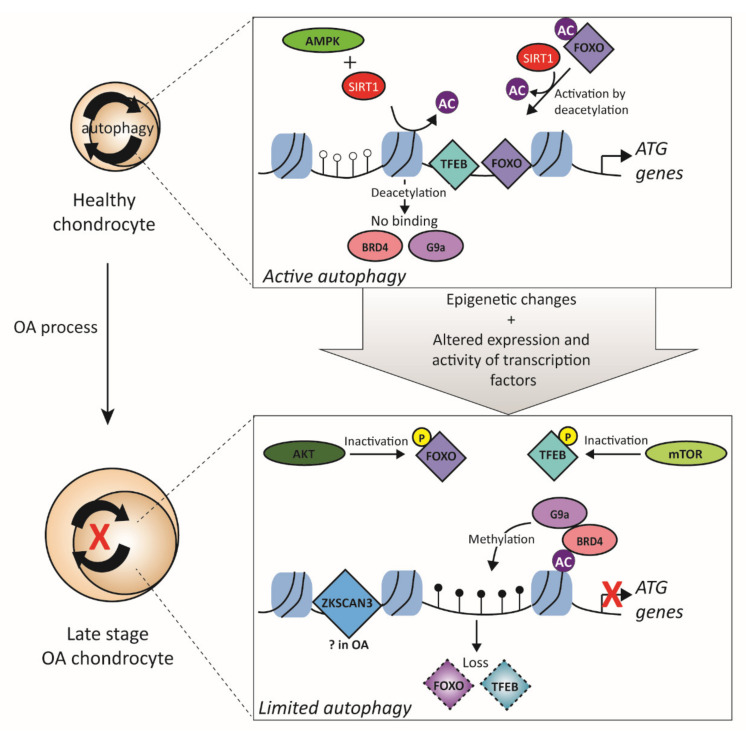
Transcription factors involved in autophagy. In a healthy chondrocyte, there is basal autophagy activity. AMPK and SIRT1 cause the release of BRD4 and the histone methyltransferase G9a via deacetylation of the histones. Furthermore, activating TFs such as TFEB can bind to the promoters of ATG genes and activate transcription. Another TF, FOXO, gets activated by deacetylation by SIRT1 and can, therefore, also activate transcription together with TFEB. In late-stage OA chondrocytes, autophagy is inhibited due to epigenetic changes and altered expression and activity of transcription factors. SIRT1 expression is downregulated in OA and, therefore, histone acetylation is not removed anymore. BRD4 binds to acetylated histone tails and recruits G9a, which hypermethylates the promoters of ATGs. Furthermore, expression of activating TFs such as TFEB and FOXO is downregulated, and their activity is also blocked by phosphorylation by AKT or mTOR. In addition, (probably), inhibitory TFs such as ZKSCAN3 are also recruited to the promoter regions. Together, this results in repression of ATGs transcription and declined autophagy in OA chondrocytes. AMPK = AMP-activated protein kinase; SIRT1 = sirtuin 1; BRD4 = bromodomain-containing protein 4; TFEB = transcription factor EB; ATG = autophagy-related; FOXO = forkhead box transcription factor class O; mTOR = mechanistic target of rapamycin kinase; ZKSCAN3 = zinc-finger protein with KRAB and SCAN domains 3.

The induction of ATG genes is, in general (thus probably also in OA), accompanied by epigenetic changes. Bromodomain-containing protein 4 (BRD4) is an epigenetic reader that recognizes acetylated histones, which acts as a repressor for ATG genes by recruiting the histone lysine methyltransferase G9a to the promoters of these genes (Figure 5) [166]. G9a hypermethylates the ATG gene promoters and thereby shuts off transcription. Importantly, BRD4 is active when there are sufficient nutrients present; however, under nutrient starvation, the AMP-activated protein kinase (AMPK), together with the deacetylase SIRT1, cause the dissociation of BRD4 from histone tails, leading to loss of its suppressor function and eventually resulting in the activation of ATG gene expression [166]. Whether BRD4 also plays a role in regulating ATG gene expression via these mechanisms in OA is not known but is likely because, in human OA knee cartilage, both mRNA and protein BRD4 levels were upregulated compared to normal cartilage, and BRD4 levels correlated positively with the severity of OA, indicating that BRD4 might also repress autophagy in OA chondrocytes [167]. Moreover, in a similar cell type as chondrocytes (nucleus pulposus cells), it was shown that inhibition of BRD4 by the protein inhibitor JQ1 resulted in enhanced autophagy [168]. Besides BRD4, SIRT1 is an important epigenetic remodeler that induces autophagy [169]. In SW1353 cells, knockdown of *SIRT1* via siRNA resulted in decreased autophagy marker expression [170]. SIRT1 exerts its function through the deacetylation of ATG proteins; however, it also promotes autophagy by activating TFs of the FOXO family [171]. As previously mentioned, SIRT1 levels are elevated in early OA chondrocytes; however, SIRT1 levels decrease with OA severity [83,85], which might partly explain the observation that autophagy is increased in early OA chondrocytes. As the process of autophagy encompasses many different genes, many different TFs are also involved in the transcriptional regulation of those genes. There are two TFs that are considered MRs of autophagy, namely, transcription factor EB (TFEB) and zinc-finger protein with KRAB and SCAN domains 3 (ZKSCAN3) [172,173]. TFEB and ZKSCAN3 have opposite functions in regulating autophagy. Overexpression of *TFEB* in HeLa cells induces autophagy [172], whereas silencing of *ZKSCAN3* also induces autophagy [173]. Both in an OA mouse model (DMM) and human knee OA cartilage, a decline in TFEB expression was observed compared to normal cartilage [174]. Furthermore, overexpression of *Tfeb* in mouse chondrocytes resulted in upregulated expression of different autophagy genes, such as *LC3*. In addition, knockdown of *Tfeb* by siRNA resulted in a decrease of autophagic flux, which is the dynamic process of forming autophagosomes and, eventually, autolysosomes, measured by LC3-II levels. TFEB is retained in the cytoplasm through phosphorylation by mTOR, leading to binding of TFEB to 14-3-3 phospho-binding proteins, which mask the nuclear localization signal, resulting in the retention of TFEB in the cytoplasm [175]. mTOR is shown to be upregulated in different animal models of OA and also in human OA cartilage [176,177]. Thus, besides the downregulated expression of TFEB in OA, its activity is also blocked by mTOR. In contrast to TFEB, ZKSCAN3 is a potent inhibitor of autophagy by repressing transcription of ATG genes such as *ULK1* and *LC3* [173]. However, the role of ZKSCAN3 in chondrocytes is not yet studied. Besides the two MRs of autophagy, other TFs also play a role in regulating the expression of ATG genes, such as different members of the FOXO TF family. FOXO TF family members, FOXO1 and FOXO3, are considered to be inducers of autophagy [156]. *FOXO1* or *FOXO1/FOXO3* knockdown via siRNA in human chondrocytes resulted in decreased expression of ATG genes in response to *tert*-butyl-hydroperoxide (tBHP)-induced oxidative stress [178]. Control transfected tBHP-stimulated chondrocytes upregulated expression of ATG genes such as *LC3* and Beclin-1, while this expression was significantly reduced in *FOXO1* and *FOXO1/FOXO3* knockdown chondrocytes. Overexpression of constitutively active forms of *FOXO* (by mutating three phosphorylation sites) resulted in higher expression of *LC3* and Beclin-1 upon tBHP stimulation in OA chondrocytes. [178]. In addition, overexpression of this mutant *FOXO1* form in healthy human chondrocytes increased the expression of *LC3* and Beclin-1 [179]. RNA sequencing revealed that levels of *FOXO* TFs were downregulated in OA cartilage compared to normal cartilage [42]. Furthermore, in both aging human cartilage and OA knee cartilage, FOXO1 and FOXO3 protein levels are reduced, together with increased phosphorylation of both FOXO TFs, resulting in cytoplasmic retention of the FOXO proteins [143]. The serine/threonine kinase AKT is one of the main kinases that phosphorylate the FOXO TFs, and the levels of activated AKT are higher in OA articular cartilage compared to normal articular cartilage [180]. Furthermore, in the DMM OA mouse model, the induction of activated AKT was observed with the progression of OA. In addition to FOXO TFs, numerous other TFs are also involved in regulating autophagy, such as p53, STAT3, NFκB, and C/EBPβ (reviewed in [156]). However, there is very little known about the role of these TFs in regulating autophagy in chondrocytes and OA.

## 4. Transcription Factors as Therapeutic Targets

**Figure 6:**
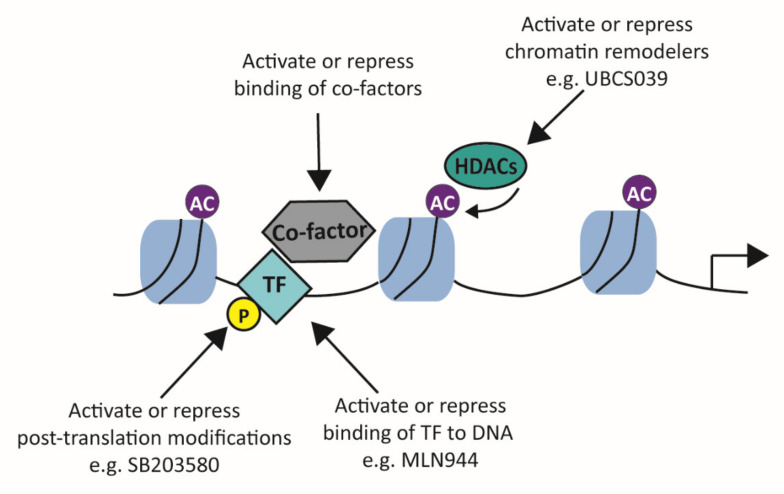
Possible therapeutic targets to regulate transcription factor function. There are different mechanisms that lead to transcription factor activation (or repression). Regulating these mechanisms can provide possible therapeutic targets. Activating or repressing the binding of cofactors, chromatin remodelers (e.g., UBCS039), and post-translational modifications (e.g., SB303580) or directly influencing the binding of transcription factors to DNA (e.g., MLN944) can be explored as therapeutic options. TF = transcription factors; HDAC = histone deacetylase.

There has been extensive research on the etiology of OA, and many attempts have been made to develop drugs to treat OA; however, none have been really successful. In the last decade, research has shifted from broad pathway analysis to more downstream signaling molecules, such asTFs, as druggable targets. A recent gain in knowledge of TF structure, function, expression patterns, and their interaction with other cofactors and the dynamics of DNA binding has helped to realize TFs as druggable targets (Figure 6). Different signaling molecules can activate different pathways that result in the use of the same TF for regulating their targeted gene expression, a phenomenon called redundancy. Therefore, targeting one signaling molecule will not necessarily diminish detrimental gene expression in a multifactorial environment such as OA. One example is that the two main ECM-degrading families, the MMPs and ADAMTSs, have some factors in their transcriptional machinery in common, such as C/EBPβ, HIF2α, and RUNX2. Targeting these transcription factors might lead to better results asmultiple ECM-degrading enzymes are then targeted instead of only inhibiting one of the members.

Furthermore, therapeutic options such as joint distraction, autologous stem cell transplantation, and even joint replacement demonstrate various degrees of clinical efficacy. One of the reasons why there is a huge variation among the efficacy of drugs or the above mentioned therapeutic options for OA is that OA is a heterogeneous disease, and, recently, it is thought that OA can be divided into different subtypes based on pathogenetic mechanisms and structural manifestations [181]. Subtyping based on the TF profile of a patient, i.e., which TFs are altered (e.g., activated or repressed), could be a valuable new way to approach treatment that has not been tried before, to our knowledge. The TF profile would give information about which key signaling pathways are activated and which process is the leading cause of OA. For example, the combination of high levels of the TFs AP-1 and RUNX2 might indicate that MMPs are the main drivers of OA, while high levels of the SOX proteins SOX4 and SOX11 might indicate more ADAMTS involvement. Subtyping based on TF profiles will help us understand the etiology of OA better and, together, will result in a more personalized medical treatment approach.

No drugs targeting transcription factors in OA are now on the market; however, transcription factors are also at the base of various other diseases such as cancer and multiple developmental disorders. A study into the role of transcription factors in human diseases implicated 164 TFs as directly linked to a human disease [182]. In the cancer field, 294 transcription factors have been found to be implicated in the disease [183], and some of these are also involved in regulating gene expression in the different processes of OA, such as JUN, SOX5, SOX9, ETS, and NFκB. MMP13, an important gene in OA development, is also implicated to play a role in the metastasis of cancer, and, here, the family of ETS transcription factors is also involved in regulation of *MMP13* expression [184]. The development of a small molecule inhibitor targeting the ETS family of transcription factors (YK-4-279) demonstrated reduced expression of *MMP13* in a prostate cell line [185]. Another anticancer drug (MLN944) prevents the binding of JUN to the AP-1 binding motif in DNA by binding to the sequence itself [186]. Furthermore, double-stranded transcription factor decoy oligodeoxynucleotides (TFD ODNs) are short pieces of double-stranded DNA that mimic the binding site of a TF and, therefore, can act as decoys for a TF [187]. In a mouse model of asthma, TFD ODNs against NFκB and STAT6 inhibited inflammation by suppressing the overexpression of proinflammatory cytokines [188]. As inflammation induced by NFκB is also involved in OA (development), this may be an interesting drug to examine in OA mouse models. In addition, NFκB plays a crucial role in SASP, and it was shown that metformin, a drug that reduces SASP and senescence, achieves this by inhibiting the TF NFκB [189]. Another option is targeting STAT3, a transcription factor downstream of IL-6 signaling, which activates the expression of different proinflammatory cytokines. Inhibition of STAT3 activity by an STA3-selective inhibitor (STA21) resulted in decreased release of proinflammatory cytokines by mesenchymal stem cells isolated from OA patients [190]. Furthermore, in the MIA rat model, STA21 caused attenuation of arthritis development by inhibiting inflammation and cartilage damage, indicating that targeting STAT3 in OA might be beneficial.

Instead of repressing a pathogenic TF, another option is either overexpressing (by pharmacologically inhibiting its degradation) or activating a TF with a positive action. SOX9, as the MR of chondrogenesis, is one of the most crucial TFs for keeping cartilage healthy, and loss of SOX9 is involved in upregulated hypertrophic differentiation, cellular senescence, and decreased ECM production. In vitro, overexpression of SOX9 in CHON-001 chondrocytes resulted in the decreased production of the inflammatory marker TNFα and decreased protein degradation of COL2A1 and SMAD3 [191]. In vivo, overexpression of *SOX9* in a surgically induced OA mice model resulted in decreased proteoglycan loss and cartilage destruction, indicating that overexpression of SOX9 might protect against OA. Injection of the another anabolic TF RUNX1 via nanomicelles in an OA mice model resulted in diminished OA development as a result of increased SOX9 and COL2A1 protein expression in the RUNX1-injected group [192], indicating that indirect activation of SOX9 might also be a therapeutic possibility.

A third option would be activating or repressing chromatin remodelers as changing chromatin accessibility is often the first step towards acquired gene expression. For example, SIRT6 was shown to be important for inhibiting cellular senescence, and expression of SIRT6 was downregulated in OA, suggesting that activating SIRT6 could suppress senescence and SASP [139]. SIRT6 activators have been developed, and UBCS039 (a specific SIRT6 activator) has shown promising results in a human cancer cell line where it triggered autophagy [193]. Additionally, in rat articular chondrocytes, it has been shown that treatment with hydroxytyrosol results in SIRT6-mediated autophagy and thereby reduces chondrocyte senescence and SASP [194], indicating that activating chromatin remodelers might be helpful in treating OA.

TFs are essential regulators of transcription in every cell type and tissue, and the same TFs can have a role in these different cells. Therefore, targeting TFs can have a major undesired impact on other tissues [195]. However, not much is known about the adverse effects of TF drugs in the clinic, as this is a recent and new field. In the cancer field, there is a lot of focus on a newly developed STAT3 inhibitor named bruceanitol. In an in-vivo study, bruceanitol was tested in a cancer mouse model via intraperitoneal injection, and no gross toxicities were determined concerning body weight; in addition, normal mouse colon tissue did not show signs of toxicity [196]. Furthermore, another TF-targeting drug, tamoxifen, which is a selective estrogen receptor modulator (SERM) that blocks ligand binding and, therefore, the conformational change necessary for the recruitment of cofactors, has been shown to be quite tissue-specific, likely due to other cofactors that are present in other cell types [197]. The SERM tamoxifen is already used in the clinic to treat breast cancer [198]. These results are promising for the development of other TF drugs. In addition, other approaches to target TFs that limit the adverse effects have also emerged, such as local injection or the use of nanodrug delivery systems ([199,200]). In OA, specific joints are often involved, which makes it likely that local injection of TF drugs can be beneficial without many side effects. The large joints, such as the knee, are excellent options for local injections, and a lot of OA patients already receive local steroid injections against the pain [201]. Another recently developed method is nanodrug delivery systems that are used to deliver drugs to a specific targeted site in a controlled manner via submicron-sized particles [202]. These particles can be modified to increase tissue retention, decrease renal clearance, or target a specific tissue. Drug delivery into the cartilage is very ineffective, and, therefore, nanodrugs may offer a solution. In a recent study, the growth factor insulin-like growth factor 1 (IGF-1) was conjugated to a nanocarrier designed to better infiltrate cartilage, and treatment of a surgical OA rat model with this IGF-1 nanodrug resulted in significantly less cartilage degeneration compared to treatment with free IGF-1 [203]. Together, these recent developments in both TF-targeting drugs and better local delivery systems are promising for the development of disease-modifying drugs for OA.

## 5. Conclusions and Future Perspectives

The most direct and effective treatment strategy for OA would be to target the transcriptional regulation machinery of genes involved in OA. The transcription factor profile, which can be used as a read-out for which signaling pathways are active, can help us understand the complex etiology of OA. In the last decade, more knowledge has been gained about TFs and how the transcriptional machinery is set in place to regulate transcription. In addition, in other fields where no therapy success is made yet with conventional targets, drug discovery is shifting towards targeting TFs. Drugs targeting TFs can possibly induce severe side effects; however, there are newly developed methods that could be used to limit this, such as a nanodrug delivery system. Further research into the method of drug delivery is needed to discover the most reliable and safe method. OA is known to be very heterogeneous, and efforts are being made to define different subtypes of OA. This transcription factor profile will help us define specific subgroups of OA and will aid a personalized treatment strategy for every patient. Of special interest are TFs that are differentially expressed in OA, but cofactor interactions and post-translational modifications might also be changed in OA. Together, these changes might explain why certain genes are up- or downregulated during OA.

## Abbreviations

ACAN	aggrecan
ADAMTS	a disintegrin and metalloproteinase with thrombospondin motif
ALPL	alkaline phosphatase
AP-1	activator protein 1
ATF2	activating transcription factor 2
ATG	autophagy-related
BAPX-1	homeobox protein Nkx-3.2
C/EBP	CCAAT-enhancer-binding protein
COL10A1	collagen type X
COL2A1	collagen type II
CREB	cAMP response element binding protein
DMM	destabilization of the medial meniscus
ECM	extracellular matrix
ENaC	epithelial sodium channel
ETS	erythroblast transformation specific
FOXA	forkhead box transcription factor class A
FOXO	forkhead box transcription factor class O
GATA4	GATA-binding factor 4
GLI	glioma-associated oncogene
HAT	histone acetyltransferase
HDAC	histone deacetylase
HIF	hypoxia-inducible factor
IHH	Indian hedgehog
IKK	IκB kinase
IL	interleukin
KCa	Ca^2+^ activated potassium channels
LC3	light chain 3
MAPK	mitogen-activated protein kinase
MEF2C	myocyte-specific enhancer factor 2C
MMP	matrix matalloproteinase
MR	master regulator
mTOR	mechanistic target of rapamycin kinase
MYOD	myoblast determination protein
NFAT	nuclear factor of activated T-cell
NFκB	nuclear factor kappa B
OA	osteoarthritis
PAX	paired box
PI3K	phosphatidylinositol 3-kinase
PITX1	paired-like homeodomain 1
PKA	cAMP-dependent protein kinase A
PTHrP	parathyroid hormone-like protein
RUNX2	runt-related transcription factor 2
SASP	Senescence-associated secretory phenotype
siRNA	small interfering RNA
SIRT1	sirtuin 1
SMAD3	SMAD family member 3
SOX9	SRY-BOX transcription factor 9
SP1	specificity protein 1
STAT3	signal transducer and activator of transcription 3
T3	active thyroid hormone
TASK-2	Acid-sensing potassium channel
tBHP	*tert*-butyl-hydroperoxide
TCF/LEF	T-cell factor/lymphoid enhancer factor
TF	transcription factor
TFD ODN	transcription factor decoy oligodeoxynucleotides
TFEB	transcription factor EB
TGFβ	transforming growth factor β
TMEM16	Ca^2+^ activated chloride channel
TNFα	tumor necrosis factor alpha
TSS	transcription start site
ULK1/2	unc-51-like autophagy activating kinase 1 and 2
ZKSCAN3	zinc-finger protein with KRAB and SCAN domain 3
